# Media influence on risk competence in self-medication and self-treatment

**DOI:** 10.3205/000214

**Published:** 2015-07-09

**Authors:** Harald Schweim, Marcela Ullmann

**Affiliations:** 1 Drug Regulatory Affairs, Rheinische Friedrich-Wilhelm-University, Bonn, Germany; 2 Committee of Research into Natural Medicines, Munich, Germany

**Keywords:** print media, internet, health education, credibility, media competence, advertising

## Abstract

Media play an important role in the reception of health risks; thus, media competence is important for enhancing the risk competence of patients and consumers. In addition to life-long health education, risk competence particularly requires careful handling of health information because, at present, the key problem is not the lack of sufficient information on health topics but the quality of such information. Patients and consumers of health procedures and health products also require information which relates to their daily life and matches their life style.

## Zusammenfassung

Medien spielen bei der Rezeption von gesundheitlichen Risiken eine wichtige Rolle. Zur Stärkung der Risikokompetenz von Patienten und Verbrauchern gehört deshalb eine Medienkompetenz. Sie erfordert neben einer lebenslangen Gesundheitsbildung besonders einen differenzierten Umgang mit Gesundheitsinformationen, weil nicht die Informationsmenge über gesundheitliche Themen derzeit ein Problem darstellt, sondern deren Qualität. Patienten bzw. Anwender gesundheitlicher Verfahren und Produkte brauchen außerdem Informationen, die sich auf ihren Alltag beziehen bzw. ihren Lebensstil treffen.

## Changes in the situation and structure of the media

In the 1990s, the internet carried only 1.0% of global communication. At the turn of the millennium, this share already amounted to more than 50.0%. Today, internet communication is estimated to carry more than 90.0% of the global information capacity [1]. Internet development was delayed in Germany in contrast to the US, but its progress was equally dynamic. Today, Germans are amongst the most intensive internet users worldwide: ‘In 2013, for instance, 77.2% of adults aged 14 years and above were online (in 2012: 75.9%). The number of internet users thus moderately increased from 53.4 to 54.2 million people. This increase was mainly based on the growing number of ‘silver surfers’ (aged 50 and above). The biggest leap from ‘offliner’ to ‘onliner’ occurred in the group of people aged 70 and above: from 20.1% in 2012 to 30.4% in 2013. Internet use increased by 6 percentage points to 82.7% in the age group of 50 to 59 years, and by 3 percentage points to 42.9% in the age group of 60 years and above. The duration of internet use has also increased considerably: In 2013, German users spent an average of 169 minutes online, which represented an increase of 36 minutes compared to the year before (in 2012: 133 minutes). The average online household has 5.3 internet-compatible devices, and their use depends on the particular user scenario. In 2012, tablet PCs were available in only 8.0% of households, and this figure rose to 19.0% in 2013. Mobile units increased the use of the internet. Mobile use increased within one year from 23.0% in 2012 to 41.0% in 2013. Apps are now used by 44.0% of German users on different devices.’[2]

The development of the internet had serious content-related as well as economic consequences for the print media. Up to the turn of the millennium, the German media had sufficient advertising potential, which provided a sound economic basis particularly for the periodical press. However, this basis was steadily decreased by the advancement of the internet. Particularly daily newspapers were affected, because a substantial part of their revenues derives from the classified section, i.e. from advertisements for real estate, used cars and jobs. Within a few years, the economic situation of most newspaper publishing houses dramatically deteriorated because of the unfavourable economic situation that had radically decreased the job market as well as the improved technological possibilities of online insertion. Furthermore, most publishing houses had to cope with continuously decreasing sale revenues and shrinking print runs, often at double-digit rates.

Most publishing houses tried to counter these challenges by introducing austerity measures, particularly in the editorial departments. Strict consolidation processes were started including the merging of editorial offices. A further austerity measure was the transformation of previously full publishing houses (producing all parts of a newspaper) either to local newspapers (using the trans-regional title page of the publishing house and only producing the local section themselves) or to local editions. This development is clearly mirrored in the list of German newspapers [3] – despite the reservations about Wikipedia as a source of science [4].

In 1968, two trans-regional newspaper publishing houses – Die Frankfurter Allgemeine Zeitung (FAZ) and Die Sueddeutsche Zeitung (SZ) – almost simultaneously started their systematic and independent news coverage of research results of natural sciences including medicine. A unit consisting of specialised journalists regularly published reports and comments on designated pages. The largely independent editors were allowed to select the content themselves. In the 1980s, most other newspapers as well as some journals followed suit and established own scientific and medical editorial offices. In the 1980s and the 1990s, nearly 1,000 scientific and medical journalists and editors were organised in the national professional associations. The first decade of the new millennium was marked by a reverse process [5] which included the streamlining of personnel as well as the marginalisation of special sections. 

## Division of the information market

At present, the area of general health communication is marked by a division of the information market. On the one hand, the market consists of a professional area in which specialist authors – who mainly work for the print media but also for health portals and reputable internet service providers – inform patients and consumers on the latest findings in the field of medicine and health. The authors usually have the appropriate qualifications, and their reports are largely based on recognised sources. The media, for which they write, are bound to the valid codes of conduct. The press code of the German Press Council contains 16 publishing principles that have to be adhered to in any type of news coverage [6]. In the case of information from the field of medicine and health, the following numbers are relevant: no. 1 ‘Veracity and the dignity of the human person’, no. 7 ‘Separation between marketing and editorial’ and particularly no. 14 ‘Coverage of medical issues’, which reads as follows: ‘In reports on medical issues, inappropriate sensational reporting should be avoided that may give rise to unsubstantiated fears or hopes of the readers. Early-stage research results should not be presented as final or nearly final.’

If a publication does not comply with the principles formulated in the press code, every citizen or organisation may make a complaint to the press council. However, the following figures show that the effectiveness of such measures has to be met with scepticism: In 2013, the press council received 1,347 complaints comprising all important subject areas. 465 of these complaints, which mostly referred to online publications, were dealt with in the relevant committee of the press council. 239 complaints were rejected as unfounded, and 226 complaints were rated as justified. However, only 28 of the 226 justified complaints were publicly reprimanded. No statistics are available on the topics of these complaints. Similar self-regulatory measures also exist in other areas of health care, for instance, for physicians or the pharmaceutical industry, but the main purpose of such measures is to avoid governmental action.

A further area of communication on medical and health-related questions is the medium internet. We analysed the search behaviour regarding the topic ‘health’ by means of 400 symptoms and 1,115 diseases and identified the average monthly search volume of the year 2013 in Germany. The 20 most frequently searched topics are listed in Table 1 (Tab. 1). The detailed analysis of the no. 1 topic ‘shingles’ showed that only 18.5% of search queries were made with keywords, whereas 81.5% of search queries were made either by means of three linked terms or a complete question. Searches by means of online search engines do not significantly differ from the peer-to-peer formats used in chat rooms or discussion forums as users obviously expect search engines to understand the semantics of the questioner. This trend is intensified by smart phone applications operated not only by a touch screen but also by voice command. However, the analysis criteria of search engines do not select sources of the best content quality but sources semantically closest to the query term.

Problems of a different nature are arising from the online exchange of information and experience of laypeople, i.e. consumer and patients. The fundamental difference between conventional media, such as print, radio and television, and internet sources is their different underlying legal framework. Internet sources often largely evade any liability. Although most participants in chat rooms and discussion forums just exchange their personal experiences, some participants also use these platforms to promote their alternative world views. The contents disseminated in this manner are not subject to any control, and the senders of such information cannot be held liable. In April 2011, a signature campaign was started against the alleged intention of the European Union to ban the sale of medicinal plants. This example shows that strong political support can be organised even by means of verifiably wrong allegations [7].

**Summary no. 1:** The current key problem is not the lack of sufficient information on health topics but the quality of such information. Effective incentives for quality are not available. Professional information providers do not have to fear any serious consequences when failing to adhere to any of the publication principles. Laypeople are neither subject to any control nor to any liability, particularly on the internet. Many examples show that patients and consumers looking for health information in Germany today have little help with separating the wheat from the chaff. [*‘On the Internet, nobody knows you're a dog’ is an adage which began as a cartoon caption by Peter Steiner and published by The New Yorker on July 5, 1993. As of 2011, this was the most reproduced cartoon from The New Yorker.*]

## Health education is a lifelong task

The way in which people absorb new information and implement such information in their behaviour mainly depends on their educational background and their methodological knowledge. The term ‘education’, coined by the German Dominican Eckart von Hohenheim in the 13^th^ century, has undergone many changes over the centuries [8]. The educational theories of the 20^th^ century showed a strong consensus that education should not just be viewed as the mere gaining of knowledge but as a process during which ‘people develop their personality by becoming independent and autonomous as well as able to solve problems and cope with life’ [9]. Thus, risk competence could be enhanced by intensifying the health education of patients and consumers of health products.

What is the current situation on health education of the general public in Germany? All pupils are taught basic health knowledge as part of their school education, and the scope and content of this health education are being widely discussed in society [10]. However, these issues will not be considered in this article. 

As a result of scientific progress, medical knowledge changes fundamentally in the period between school years and mid-life, when health topics usually become more relevant. Therefore, even extensive school health education on its own cannot be the basis of sufficient risk competence in later life. For this reason, health education of adults is very important, and health education is a lifelong task. 

Adult health education in Germany is provided by two completely different sectors: the public law sector and the private sector. The most important institutions of the public law sector are the adult educational centres established in 1947. The German Adult Education Association (Deutscher Volkshochschul-Verband – DVV) currently comprises 924 adult educational centres with over 3,000 branches. More than 50% of these centres are managed by towns, rural districts and municipalities. According to their own information, these centres derive around 40% of their funding from participants’ fees, and the remaining amount is provided by a variety of public sources. 

The educational opportunities cover a wide range of topics including health education and prevention. However, the adult educational centres entered into an exclusive cooperation on health education with a private information provider, the publishing house ‘Wort&Bild’ in Baiersbrunn, in June 2012. This cooperation blurs the otherwise clear distinction between the public educational mandate and economically motivated communication. The basis is a cooperation agreement in which the management of the publishing house ‘Wort&Bild’ and the German Adult Education Association agreed on a long-term collaboration on various topics, including the use of a well-known pharmacy customer magazine and other print materials [11]. Analogous to the adult educational centres, several other public and semi-public institutions offer health information and health education in the context of adult education in Germany:

- Aktionsforum Gesundheitsinformationssystem (afgis) e.V.
  http://www.afgis.de
- BAG-Patientinnenstellen
  http://www.gesundheits.de/bagp/bagp_prima.html
- Beratungsstellen in Deutschland zu Gesundheitsthemen
  http://www.bzga.de/?uid=423d126a8f206d187b4393b03d5fcc29&id=beratung
- Bundesvereinigung Prävention und Gesundheitsförderung e.V. (BVPG) / Federal Association of Prevention and Health Promotion
  http://www.bvpraevention.de
- Bundeszentrale für gesundheitliche Aufklärung (BZgA)/Federal Centre for Health Education
  http://www.bzga.de
- BZgA: Katalog für Audiovisuelle (AV) Medien zu Gesundheitsförderung und Prävention
  http://www.bzga-avmedien.de
- Deutsche Gesundheitshilfe e.V. – Bundesgeschäftsstelle (DGH)
  http://www.gesundheitshilfe.de
- evb-online.de – Ernährung- und Verbraucherbildung im Internet
  http://www.evb-online.de/
- FLUGS – Fachinformationsdienst Lebenswissenschaften, Umwelt und Gesundheit
  http://www.helmholtz-muenchen.de (publication ceased)
- optipage – Informationsportal zur Gesundheit
  http://www.optipage.de/gesundheit.html
- Patienten-Information.de – Patientenschulungen
  http://www.patienten-information.de
- Robert Koch-Institut (RKI)
  http://www.rki.de
- UGB – Unabhängige Gesundheitsberatung
  http://www.ugb.de
- Verzeichnis der Bundesvereinigung und der Landesvereinigungen für Gesundheitsförderung
  http://www.gesundheit-nds.de/index.php/medien/links

Next to the public sector of adult education, a commercial sector of health education providers has developed, but its extent is difficult to assess. Most enterprises offer courses on specific topics, for instance, on medicinal plants or nutrition, and the knowledge gained by attending these courses is supposed to improve the health or quality of life of the participants. Many institutes declare their health education courses as specialist training, but course attendance does not entitle participants to practice any type of medicine. Schools for alternative practitioners – funded either by associations or individuals – can be found throughout Germany; because of the high number of annual graduates, these schools play an important role as mediators of health knowledge. The number of participants of such courses is difficult to assess because no reliable statistics is available, neither regarding the number of schools nor the size of the classes. Most schools mainly teach complementary medicine or just one of its subsections.

A wide variety of health information is also provided by specialised medical books and health guides. Next to periodicals, specialised medical books or health guides are an important part of the print media sector. Particularly health guides, which are often sold in millions, are a big market success and thus an important factor in health education. The price for such publications is often much higher than 10 Euros. The amount of 10 Euros corresponded to the practice fee patients had to pay until December 2012 when consulting their general practitioner, and this fee was considered to be a tool for controlling the health behaviour of patients. The fact that patients and consumers were prepared to pay this kind of money for health information shows that such information is considered highly valuable. 

Health guides cover a wide range of topics and are often professionally done and didactically well structured. However, health guides are not medical books and are mainly intended to meet the expectations of their readers. Health guides are a product that needs to be sold; thus, their priority is neither serious information nor scientificity, as can be seen in publishing programmes.

**Summary no. 2:** Health education is a lifelong task, if only because of the continuously increasing knowledge in the field of medicine. Next to the public sector in Germany, a commercial sector of health education providers has developed that is not subject to any content or quality criteria. At present, neutrally presented health information is hardly available to consumers or patients. Even institutions claiming to be critical observers of the medical field or consumer advisors are sometimes motivated by self-interest, as can be seen in the example of the adult educational centres.

## Information behaviour depends on age

What sources of information patients and consumers of health products use and questions regarding the credibility of individual information providers are often investigated by market research institutes. Between 2005 and 2009, the market research and consulting institute ‘Psychonomics AG’ in Cologne conducted representative annual surveys using different medical and health-related focal points. The last survey in 2009 focussed on the information behaviour of internet users [12]. Altogether 2,000 internet users aged 16 years and above were questioned, and the results of the survey were representative. The survey showed that 79% of the respondents searched for information on health topics on the internet, 72% asked physicians, 64% read pharmacy customer magazines and 60% watched health programmes on television or read reports in daily newspapers and magazines. 

In 2009 and 2013, the market research institute ‘midline media GmbH’ in Berlin conducted a study on the topic ‘A trend comparison of health and medicine’. Both studies had a similar design, allowing not only the assessment of the current situation but also a detailed statement about the longer-term trend [13]. Each study included a telephone survey of randomly chosen people aged 14 and above (stratified multistage sample); in 2009, the responses of 1,001 people were evaluated and in 2013 the responses of 508 people. The trend comparison showed television as the most important source of information in the mass media (61% in 2013 vs. 66% in 2009) followed by pharmacy customer magazines (53% in 2013 vs. 56% in 2009) and the internet (40% in 2013 vs. 34% in 2009). The importance of newspapers and magazines dropped from third place in 2009 (51%) to fifth place in 2013 (33%). In the context of personal communication, the importance of medical consultation by physicians increased from 64% in 2009 to 71% in 2013. The importance of family members and friends as advisors on health topics decreased from 52% in 2009 (ranking second) to 40% in 2013.

An analysis differentiated according to the age of the respondents showed some common elements as well as the following differences: The internet was used by 57% of people aged between 14 and 49 years, by 31% of people aged between 50 and 59 years, but only by 16% of people aged 60 years and above. The reverse situation could be seen with regard to pharmacy customer magazines which were read by only 29% of people aged between 14 and 29 years, by 56% of people aged between 50 and 59 years but by 68% of people aged 60 years and above. A further difference between the younger and older age groups was that younger people aged between 14 and 29 years and between 30 and 49 years attached much more importance to the recommendation by family members and friends (46% and 48% respectively) than older people (31%). Nearly 40% of younger people considered books, magazines and the internet as trustworthy in contrast to 10% and 16% of people aged over 60 years. Thus, different content presentation in information sources results in different age-specific estimation of risks and advantages of health measures and medicinal products.

**Summary no. 3:** In Germany, younger and older people show significant differences when searching for health information. The different age groups show typical generation-specific behavioural patterns. Classifying the credibility of individual sources also depends on the respective generation. When searching for health information, younger people use the internet more often than older people and also more often rely on the information offered. This situation is reversed with regard to pharmacy customer magazines, which are read by two thirds of people aged 60 years and above but barely used by the younger generations. Different assessment of the content of individual information sources also leads to different assessment of the risks and benefits of health measures and medicinal products. Physicians are the most important and most reliable source of health information for most Germans, independent of their age.

## Improved media competence also increases the risk competence of patients and users

Information on new diagnostic and therapeutic possibilities, new scientific findings and risks or health hazards warnings vary in efficacy with regard to individual perception and the assessment of health risks. The following statement basically applies: ‘People are more afraid of dangers which they do not know from their own experience or which they cannot directly perceive in a sensual manner’ [14]. Thus, an exaggerated positive picture of new medical treatments or findings generally creates fewer problems than the misinterpretation or isolated presentation of risks and side effects of medical measures. Wrong expectations towards a new or an alternative therapeutic option rarely bears a risk for the affected person, for instance, because of rejecting a well-established method of treatment. In general, physicians are considered trustworthy enough (see above) to correct such misinterpretations, particularly if patients are in continuous contact with their physician. The reception of information is more difficult in the case of health topics without any expert consensus. Such lack of consensus is more frequent than is generally assumed, particularly in the case of methods for which experts consider practical experience insufficient, for instance, proton therapy in cancer treatment. In this case, patients have to rely on their own competence when making a decision, which often means that they do not only have to bear the risk of the decision but also the subsequent costs.

In daily life, negative headlines regarding real or supposed health hazards are often more problematic than unreasonably positive reports. The reinforcement of health risks through the media – particularly when vividly described – has become a recognised fact. Many examples show how media reporting unduly raised public attention because a topic was presented as a serious and acute risk. For instance, reports on the danger of an outbreak of the Creutzfeldt-Jakob disease resulted in hysterics that lasted several weeks. The resulting campaign was also joined by renowned newspapers such as the Frankfurter Allgemeine Zeitung [15]. Another example is the long-lasting discussion about the alleged harmfulness of amalgam in dental fillings which even resulted in a court judgement ordering a scientific investigation into the matter [16]. Benzpyrenes in barbecue meat or dioxin in the environment present similar examples. These examples show how the transmission of correct but isolated findings of cellular and animal models to humans may lead to wrong conclusions and fears. The examples also show how substances may become classified as dangerous, although their concentrations in reality cannot be compared with those in the laboratory. Additional problems arise if exaggerated fears of real health risks in daily life, for instance, smoking, may distract attention from other factors, such as high-calorie diets or lack of physical activity.

**Summary no. 4: **The perception of potential dangers is naturally subject to bias which often results in underestimating known and day-to-day risks and overestimating rare events. Media reporting usually increases the overestimation of risks. In order to avoid such misconceptions, people should develop a critical state of mind in their dealings with the media. With regard to health issues, risk competence furthers the routine use of different information sources and the assessment of their contents. Patients and consumers of health measures and procedures require information which relates to their daily life and matches their life style. Even seriously ill patients wish for additional information on how to conduct themselves in their daily life over the course of the treatment. In this respect, however, numerous knowledge gaps exist in the field of medicine which are often exploited by self-appointed but poorly informed ‘experts’.

## Advertising is a special form of communication

In Germany, the advertising of medicinal products and treatment options is regulated by the law on the advertising of medicines (Heilmittelwerbegesetz – HWG) [17]. Prescription medicines and treatments are not examined here; we want to concentrate on the advertising of over-the-counter drugs and nutritional supplements – thus food products with a special focus; such supplements termed nutraceuticals are not subject to the law on the advertising of medicine but to food safety legislation [18]. [*Advertising statements and promises about nutraceuticals are regulated in the EU directive no. 1924 of 2006 (Health Claims). Similar to other food products, statements on diseases or indications are not permitted.*]

In principle, advertising can be defined as the dissemination of information for the purpose of sale or promotion of use and also tends to be perceived in this manner by consumers. In Germany, the credibility of advertising statements about over-the-counter drugs and treatment methods which are not excluded from advertising is based on paragraph 3 of the law on the advertising of medicines. This paragraph not only prohibits misleading advertisements but also shows such deceptions in detail so that blatant exaggerations in this area have been mostly prevented so far. With regard to over-the-counter drugs, problems resulting from advertisements mostly ensue from a regulation that prohibits the reporting of scientific research results related to a specific drug. However, generic statements are often misleading, particularly in the area of natural medicinal products. For instance: For a clinically investigated valerian product, the following wording is not allowed: ‘The clinical study conducted by the university xx showed that sleep disorders decreased by yy% in zz% of patients treated with the ethanoic valerian extract for four weeks.’ In contrast, valerian products that are not clinically investigated may be advertised with the following wording: ‘Valerian has proven successful in the treatment of sleep disorders.’ Such regulations inevitably lead to misunderstandings.

Misunderstandings in the area of nutraceuticals mainly arise because these substances are often seen as medicinal products. A significant difference between medicinal products and food products is the basic assumption that – in order to be marketable – medicinal products require a positive benefit-risk ratio. Furthermore, medicinal products are subject to continuous risk monitoring. By definition, food products are free of risk and thus not subject to any risk monitoring. Particularly in the area of products based on medical herbs (termed ‘botanicals’), food products (nutraceuticals) and medicinal products on the market often contain the same plant as a raw material, and the health-related statements of these products are often rather similar [19]. Thus, the different character of these two product groups remains unclear. Laypeople do not differentiate between product category and other marketable categories because they are interested in the benefits of a product, which may even lead to further misinterpretation. In advertising, such wrong interpretations are desired and often used deliberately.

**Summary no. 5:** Consumer competence would be effectively enhanced by regulating the declaration of natural medicinal products independent of the product category ‘medicinal product‘ or ‘food product‘ as well as by coordinated advertising regulations.

## Notes

### Competing interests

The authors declare that they have no competing interests.

### Remarks to this article

This article is part of an extensive multipart publication on the topic ‘Enhancing risk competence’ which is being compiled by the interdisciplinary work group of the same name. This part only deals with the general media and the communication with lay people and patients. The medical press and the communication between experts (physicians, pharmacists and medical personnel) are subjects of another part of this multipart publication.

## Figures and Tables

**Table 1:**
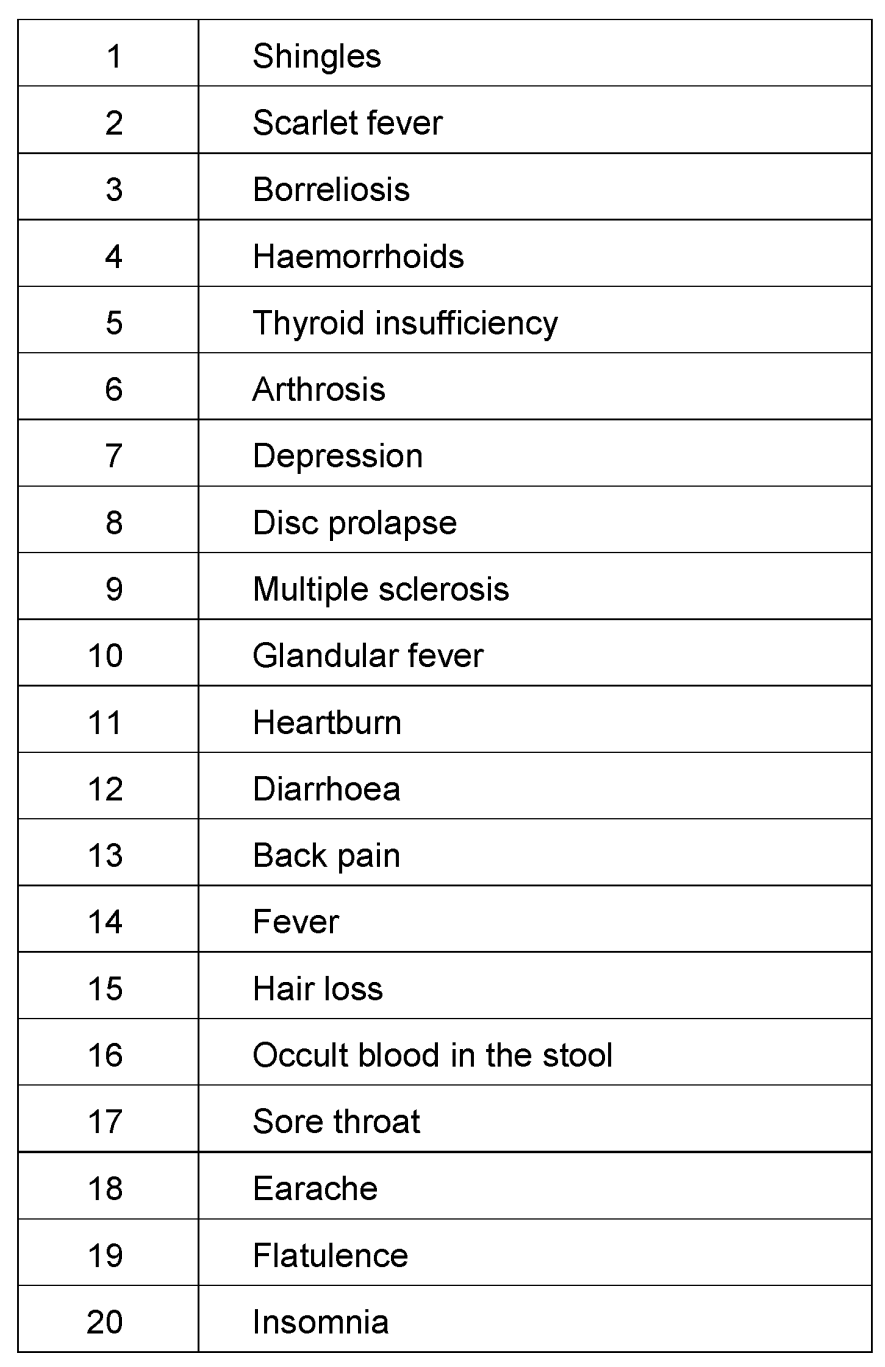
The most frequently searched health topics on the internet in 2013
